# Supplementary material for: Molecular correlates of muscle spindle and Golgi tendon organ afferents
Source: Nat Commun. 2021 Mar 1;12:1451. doi: 10.1038/s41467-021-21880-3 (PMC7977083; doi:10.1038/s41467-021-21880-3)
Supplement: Supplementary file 1 — Supplementary Information [file 41467_2021_21880_MOESM1_ESM.pdf]

Supplementary Figure 1

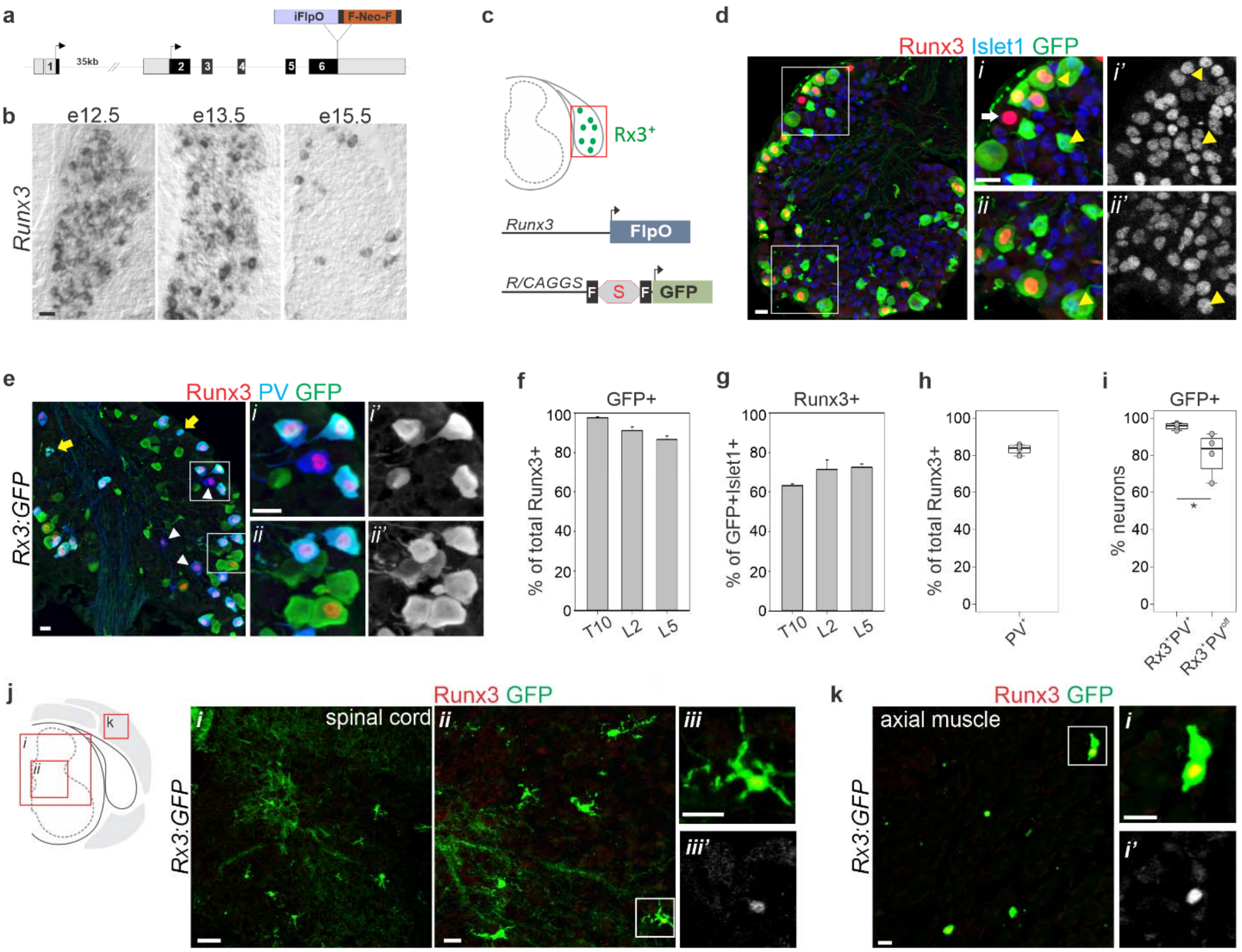

Figure S1. Phenotypic analysis of *Rx3:FlpO* mice.

a. Diagram depicting the localization of the FlpO recombinase coding sequence within the *Runx3* genomic locus. Boxed areas indicate *Runx3* coding (in black) and non-coding (in gray) exons; the two Runx3 start sites are indicated (black arrows). An IRES-FlpO-neo cassette was inserted just after the Runx3 stop codon through homologous recombination in mouse embryonic stem cells. See Methods for details.

b. Expression of *Runx3* (as assessed by RNA *is situ* hybridization) during early developmental stages indicates the gradual restriction of *Runx3* transcript to proprioceptive sensory neurons.

c. Illustration of genetic strategy to label Runx3^{+} DRG sensory neurons in *Rx3:FlpO;RCE* mice (*Rx3:GFP*).

d. Expression of Runx3, Islet1, and GFP in p2 lumbar DRG of *Rx3:GFP* mice. Boxed areas are enlarged in (*i, ii*). Corresponding images (*i', ii'*) show expression of Islet1 (Isl1). Few Rx3^{+} neurons do not label with GFP (arrow), while GFP is observed in a substantial number of Rx3^{off}Islet^{+} neurons (arrowheads).

e. Expression of Rx3, PV, and GFP in p2 L2 DRG of *Rx3:GFP* mice. Boxed areas are enlarged in (*i, ii*). Corresponding images (*i', ii'*) show expression of GFP. Few $Rx3^+PV^+$ neurons do not label with GFP (arrowheads). While fragments of GFP^+PV^+ neurons (lacking a nucleus) can be detected (arrows), 'full size' (nucleated) GFP^+PV^+ neurons that lack Rx3 are not observed.

f. Percentage of $Rx3^+$ neurons co-expressing GFP (of all $Rx3^+$ neurons) in T10, L2 and L5 ganglia of p2 *Rx3:GFP* animals. Mean percentage (± S.E.M.) for T10: 97.6 ± 0.5%, n = 2 DRG (DRG_1: 15 sections, DRG_2: 13 sections); L2: 91.2 ± 1.9%, n = 6 DRG (DRG_1: 12 sections, DRG_2: 12 sections, DRG_3: 15 sections, DRG_4: 11 sections, DRG_5: 11 sections, DRG_6: 13 sections); L5: 86.6 ± 1.8%, n = 2 DRG (DRG_1: 20 sections, DRG_2: 21 sections).

g. Percentage of $Islet1^+GFP^+$ neurons co-expressing Runx3 in T10, L2 and L5 ganglia of p2 *Rx3:GFP* animals. Mean percentage (± S.E.M.) for T10: 63.1 ± 0.6%, n = 2 DRG; L2: 71.6 ± 3.4%, n = 2 DRG; L5: 72.6 ± 1.2%, n = 2 DRG. (Number of sections/DRG as in 1f, minus $DRG_{1\text{-}4}$ for L2). (When combined, 30.9 ± 2.1% of $Islet1^+GFP^+$ neurons lack expression of Rx3; n=6 DRG.)

h. Percentage of $Rx3^+PV^+$ and $Rx3^+PV^{off}$ neurons (of all $Rx3^+$ neurons) in L2 DRG of p2 *Rx3:GFP* animals. Mean percentage for $Rx3^+PV^+$: 83.4 ± 1.3%, n = 4 DRG (DRG_1: 12 sections, DRG_2: 12 sections, DRG_3: 15 sections, DRG_4: 11 sections). Boxes show the median, 25th, and 75th percentile, and whiskers extend to the most extreme data point less than 1.5 times the interquartile range.

i. Percentage of GFP^+ neurons of all $Rx3^+PV^+$ or $Rx3^+PV^{off}$ neurons in L2 DRG of p2 *Rx3:GFP* animals. Mean percentage for $GFP^+Rx3^+PV^+$: 95.7 ± 0.9%, n = 4 DRG; $GFP^+Rx3^+PV^{off}$: 81.0 ± 5.8%, n = 4 DRG. (Number of sections/DRG as in 1h). $Rx3^+PV^+$ neurons are slightly more likely to induce GFP expression than $Rx3^+PV^{off}$ neurons (p=0.046, two-tailed t-test). Boxes show the median, 25th, and 75th percentile, and whiskers extend to the most extreme data point less than 1.5 times the interquartile range.

j. Expression of Runx3 and GFP in p12 spinal cord of *Rx3:GFP* animals. GFP is observed in central collaterals of pSNs as well as in a small number of cells that resemble microglia. No Rx3 or GFP expression was detected in spinal inter- or motor neurons. Images *i*, and *ii* are taken from corresponding spinal areas indicated in schematic. Boxed area in *ii* is shown at higher magnification in *iii* and *iii'.*

k. Expression of Runx3 and GFP in p12 axial muscles of *Rx3:GFP* animals. GFP (and Rx3) is observed in small numbers of cells that resemble muscle satellite cells. Image taken from corresponding muscle area indicated in schematic of j. Boxed area is shown at higher magnification in *i* and *i'.*

Data obtained from at least three (a, d, e) and two (j, k) biological replicates. Scale 10 μm (b) and 20 μm (d, e, j, k).

Supplementary Figure 2

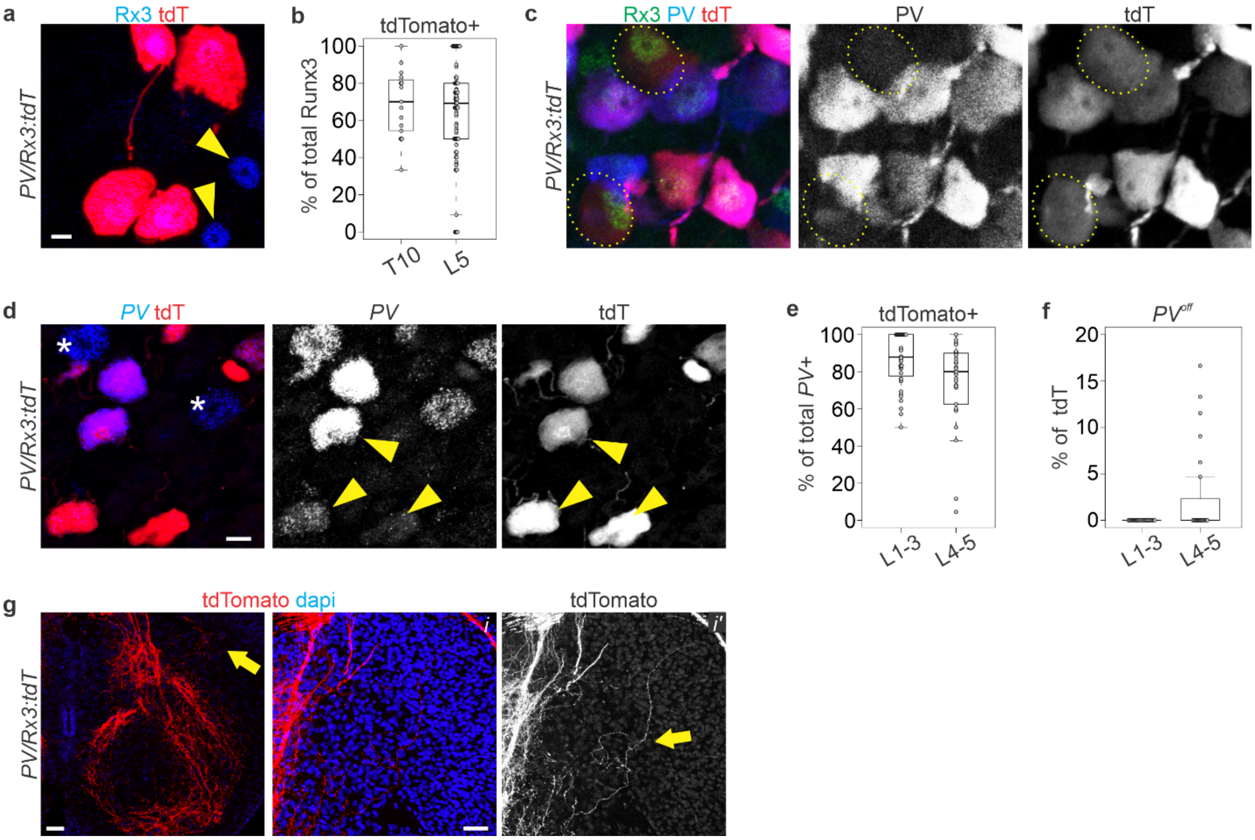

Figure S2. Phenotypic analysis of *PVRx3:tdT* mice.

a. Expression of Runx3 and tdT in lumbar DRG of *PVRx3:tdT* animals. tdT^+ neurons invariably express Rx3. $Rx3^+tdT^{off}$ neurons (arrowheads) correspond to PV^+Rx3^+ neurons in which Cre/Flp recombination failed, or to $Rx3^+PV^{off}$ neurons.

b. Percentage of tdT^+Rx3^+ neurons (of all $Rx3^+$ neurons) in T10 and L5 ganglia of p28 *PVRx3:tdT* animals. Mean percentage for T10: 69.0 ± 4.4%, n = 17 sections; L5: 64.2 ± 2.9%, n = 83 sections. Boxes show the median, 25th, and 75th percentile, and whiskers extend to the most extreme data point less than 1.5 times the interquartile range.

c. Expression of PV, Rx3, and tdT in lumbar DRG of p12 *PVRx3:tdT* mice. Some $Rx3^+tdT^+$ neurons express barely detectable levels of PV (dotted circles), consistent with observations in (d).

d. Expression of *PV* and tdT in lumbar DRG of adult (≥p56) *PVRx3:tdT* animals. Nearly all tdT^+ neurons express *PV*, but the level of *PV* transcript is highly variable (compare neurons indicated by arrowheads). *PV*$^+$tdT^{off} neurons (asterisk) correspond to PV^+Rx3^+ neurons in which Cre/Flp recombination failed, or to

PV^+Rx3^{off} rapidly- adapting (RA) cutaneous mechanoreceptive neurons (e.g. Meissner, Pacinian, or Lanceolate afferents).

e. Percentage of tdT^+*PV^+* neurons (of all *PV^+* neurons) in rostral (L1-3) and caudal (L4-5) lumbar DRG of adult (≥p56) *PVRx3:tdT* animals. Mean percentage for L1-3: 86.9 ± 2.1%, n = 44 sections; L4-5: 73.9 ± 4.2%, n = 30 sections. Consistent with observations that RA mechanoreceptors are more prevalent at caudal lumber levels, the percentage of tdT^+*PV^+* neurons is higher at the L1-3 segmental level compared to L4-5 levels (p=0.004; two-sided Mann-Whitney rank sum test). Boxes show the median, 25th, and 75th percentile, and whiskers extend to the most extreme data point less than 1.5 times the interquartile range.

f. Percentage of tdT^+*PV^{off}* neurons (of all tdT neurons) in rostral (L1-3) and caudal (L4-5) lumbar DRG of adult (≥p56) *PVRx3:tdT* animals. Mean percentage for L1-3: 0.8 ± 0.8%, n = 31 sections; L4-5: 2.7 ± 1.1%, n = 23 sections. Boxes show the median, 25th, and 75th percentile, and whiskers extend to the most extreme data point less than 1.5 times the interquartile range.

g. Expression of tdT in MS and GTO afferent collaterals in spinal cord of p12 *PVRx3:tdT* mice. Higher magnification of the dorsal horn in (*i, i'*) suggest that some tdT^+ axons (arrow in *i'*) enter the spinal cord laterally.

Similar data obtained from at least three (a, c, d) and two (g) biological replicates. Scale 10 μm (a,c,d) and 50 μm (g).

Supplementary Figure 3

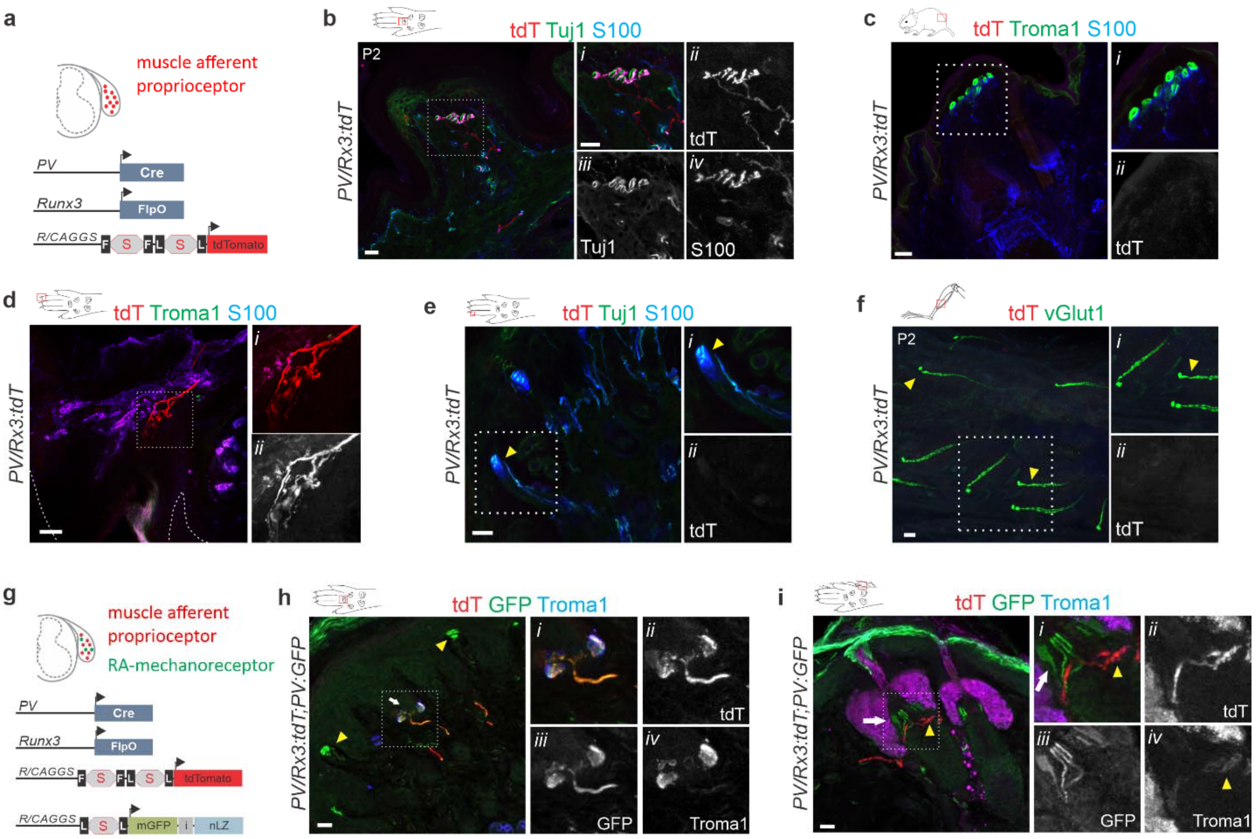

Figure S3. Analysis of cutaneous afferents in *PVRx3:tdT* mice.

a. Illustration of genetic alleles enabling the selective labeling of $PV^{+}Rx3^{+}$ proprioceptive muscle afferents in DRG.

b. Expression of tdT, TuJ1 and S100 in forelimb glabrous skin of p2 *PVRx3:tdT* mice indicates association of tdT^{+} axons with TuJ1 labelled Merkel cells. Boxed area is shown at higher magnification in (*i-iv*).

c. Expression of tdT, Troma1 and S100 in (hairy) back skin of adult (≥p56) *PVRx3:tdT* mice. In contrast to glabrous fore paw skin, innervation of Troma1 Merkel cells in back (hairy) skin was never observed. Boxed area is shown at higher magnification in (*i-ii*). Schematic obtained from kindpng.com.

d. Expression of tdT, Troma1, and S100 in forelimb digit nail of ≥p56 *PVRx3:tdT* mice. Boxed area is shown at higher magnification in (*i-ii*).

e. Expression of tdT, TuJ1 and S100 in forelimb glabrous skin of p2 *PVRx3:tdT* mice indicates absence of tdT^{+} axons from Meissner corpuscles (arrowheads). Boxed area is shown at higher magnification in (*i-ii*).

f. Expression of tdT and vGlut1 in the interosseous membrane of p2 *PVRx3:tdT* mice indicates absence of tdT^{+} axons from Pacinian corpuscles (arrowheads). Boxed area is shown at higher magnification in (*i-ii*).

Schematic from “The anatomy of the Laboratory Mouse” (by M. J. Cook, Elsevier, 1965) and obtained through the web implementation of the book at http://www.informatics.jax.org/cookbook/.

g. Illustration of genomic alleles enabling the selective labeling of PV^+Rx3^+ muscle proprioceptor and PV^+Rx3^{off} low threshold cutaneous mechanoreceptors in DRG.

h. Expression of tdT, GFP and Troma1 in forelimb skin of ≥p28 *PVRx3:tdT*; *PV:GFP* mice showing tdT^+GFP^+ axons associated with $Troma1^+$ Merkel cells (arrow). No tdT^+ axons associate with GFP^+ Meisner corpuscles (arrowhead). Boxed area is shown at higher magnification in (*i-iv*).

i. Expression of tdT, GFP and Troma1 in forelimb skin of ≥p28 *PVRx3:tdT*; *PV:GFP* mice simultaneously showing tdT^+GFP^{off} axons associated with $Troma1^+$ Merkel cells around a hair follicle (arrowhead), and $tdT^{off}GFP^+$ longitudinal Lanceolate endings (arrow). Boxed area is shown at higher magnification in (*i-iv*). We postulate that the mosaic activation of GFP in Merkel cell afferents may result from a) low *PV* transcript levels in these afferents, and from b) a higher Cre-mediated *loxP* recombination efficiency in the *Ai65:tdTomato* reporter when compared to the *PV:GFP* reporter.

Similar data obtained from at least three (a, c, d) and one (h, i) biological replicates. Scale 20 μm.

Supplementary Figure 4

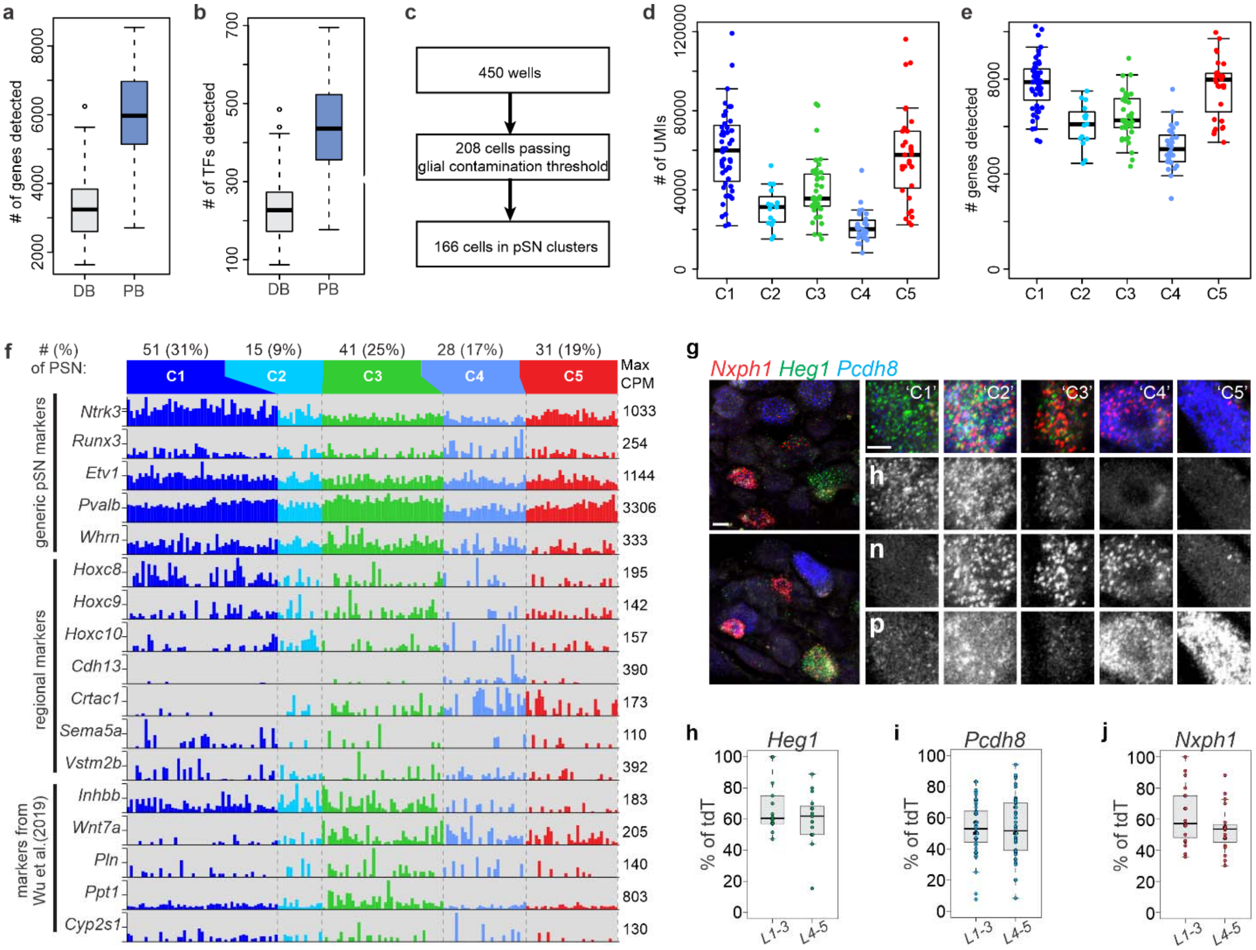

Figure S4. Analysis of pSN single cell RNA sequencing data.

a, b. Number of detected genes (all) (a) and number of detected transcription factors (TFs) (b) obtained for adult proprioceptors using either droplet-based (DB) or plate-based (PB) single cell sequencing platforms, using only the common genes in both data set transcriptome versions (to account for differences in total genes based on version differences). Detection was defined as a count value > 0. The number of detected genes (a) and transcription factors (b) per neuron is significantly higher for the plate-based sequencing method. (Ratio of mean genes detected in plate-seq vs. droplet (all genes): 1.81, DB mean (median) number of detected genes: 3304 (3240), PB mean (median) number of detected genes: 5989 (5968); p = 4.15e-55, two-sided Mann-Whitney test.) (Ratio of mean TFs detected in plate-seq vs. droplet (TFs only): 1.90, DB mean (median) number of detected TFs: 231 (227), PB mean (median) number of detected TFs: 439 (436); p = 9.25e-52, two-sided Mann-Whitney test.) DB-seq. data obtained from Sharma et al., 2020; PB-seq. data obtained from this study. Boxes show the median, 25th, and 75th percentile, and whiskers extend to the most extreme data point less than 1.5 times the interquartile range.

c. Overview of the number of starting and filtered cells profiled using the plate-seq method (see Methods). Of the 450 initial plate wells, 208 cells passed the threshold for minimal cross-talk with non-neuronal transcripts, chiefly satellite cell genes such as Mbp (see Methods). Of these 208 cells, 166 were determined to be in the five clusters described in the main text, whereas the remainder fell into non-proprioceptor classes, or were in clusters with fewer than 10 cells.

d, e. Boxplots showing the number of (d) Unique Molecular Identifiers (UMIs) and (e) genes detected per cell (n=51 for C1; 15 for C2; 41 for C3; 28 for C4; 31 for C5), organized by cluster. Points for each individual cell are overlaid on the boxplots. Boxes indicate medians and $25^{th}/75^{th}$ percentiles, and whiskers extend to the furthest point less than 1.5 standard deviations from the mean.

f. Barplots showing expression (counts per million, CPM) of previously reported pSN and regional markers, with individual cells arranged by cluster. The maximum CPM for each gene is reported on the right hand side.

g. Expression of previously identified pSN subset markers, *Heg1* (h), *Nxph1* (n), and *Pcdh8* (p), in lumbar DRG of adult (≥p56) *PVRx3:tdT* mice. Individual neurons ('C1'-'C5'; not part of larger images) serve as putative examples for the different classes of neurons uncovered through single cell RNAseq analysis. Similar data obtained from at least three biological replicates.

h-j. Percentage of *Heg1*$^{+}$tdT^{+} (h), *Pcdh8*$^{+}$tdT^{+} (i), and *Nxph1*$^{+}$tdT^{+} (j) neurons (of all tdT^{+} neurons) in rostral (L1-3) and caudal (L4-5) lumbar DRG of adult (≥p56) *PVRx3:tdT* animals. Mean percentage for *Heg1* (L1-3): 67.4 ± 4.4%, n = 14 sections; *Heg1* (L4-5): 59.8 ± 4.5%, n = 15 sections; *Pcdh8* (L1-3): 53.5 ± 2.3%, n = 50 sections; *Pcdh8* (L4-5): 54.4 ± 3.0%, n = 44 sections; *Nxph1* (L1-3): 62.3 ± 4.5%, n = 19 sections; *Nxph1* (L4-5): 53.5 ± 3.3%, n = 20 sections. Boxes show the median, 25th, and 75th percentile, and whiskers extend to the most extreme data point less than 1.5 times the interquartile range.

Scale 10 μm.

Supplementary Figure 5

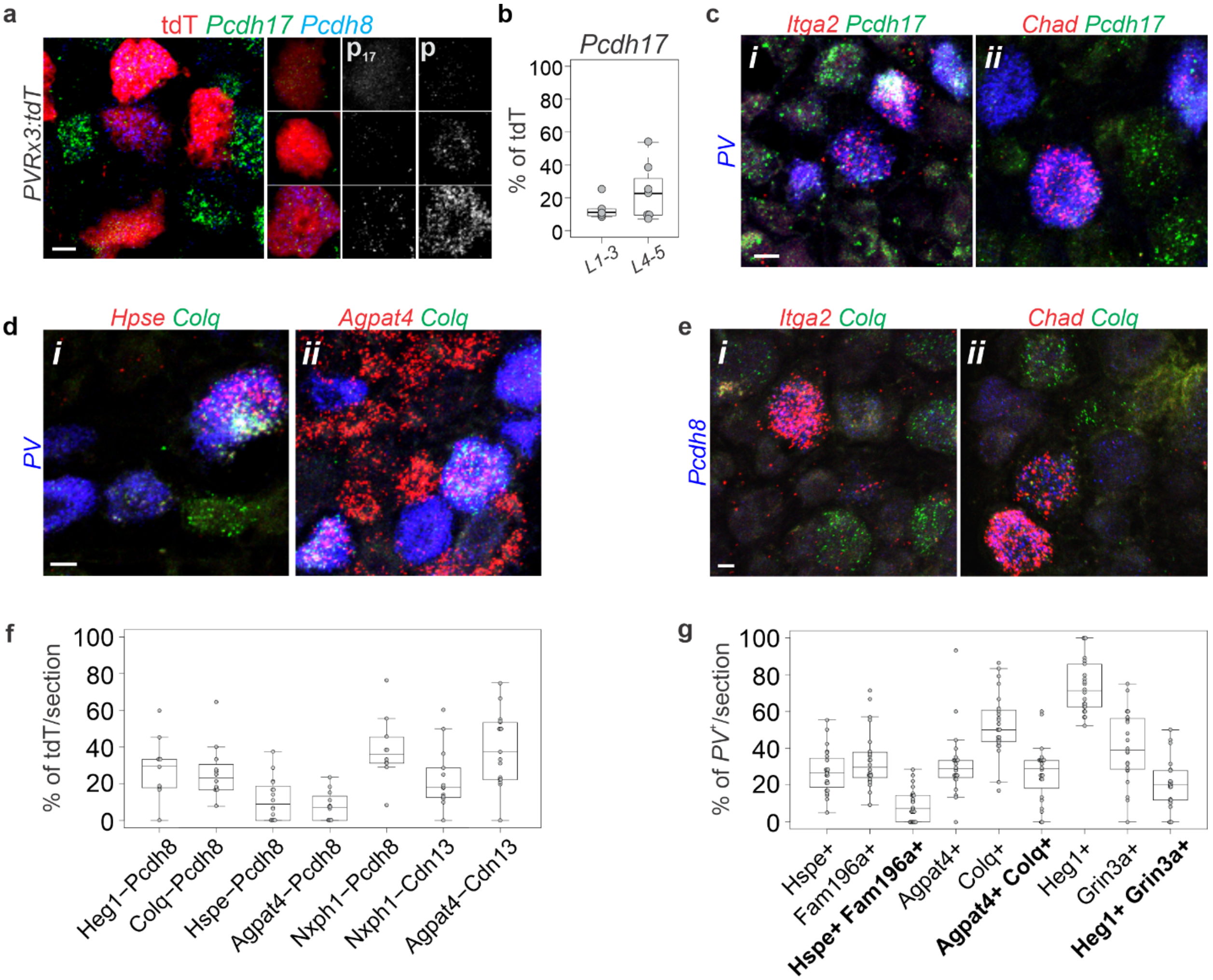

Figure S5. Validation of transcriptionally distinct pSN clusters.

a, b. Expression of the cluster 5 transcripts *Pcdh17* and *Pcdh8* in ≥ p56 lumbar DRG of *PVRx3:tdT* mice. Images of individual neurons represent examples of observed transcript combinations other than those observed in the main image. (a) Pcdh17 (P_{17}) transcript is generally only observed in tdT^+ neurons with high levels of *Pcdh8* (p) transcript expression. (b) Percentage of *Pcdh17*$^+$$tdT^+$ neurons (of total tdT^+) in ≥p56 rostral (L1-3) or caudal (L4-5) lumbar DRG of *PVRx3:tdT* animals. Median percentage ± S.E.M. at L1-3: 13.4 ± 3.0%, n=5 sections; L4-5: 23.8 ± 6.6%, n=7 sections. Boxes show the median, 25th, and 75th percentile, and whiskers extend to the most extreme data point less than 1.5 times the interquartile range.

c. Expression of cluster 5 transcripts *Itga2*, *Pcdh17*, and *Chad* in *PV*$^+$ neurons in ≥ p56 lumbar DRG of wild type mice. Expression of *Pcdh17* is observed in many neurons in DRG, but within *PV*$^+$ neurons localizes to *Itga2*$^+$ (*i*) or *Chad*$^+$ (*ii*) PV neurons.

d. Expression of cluster 1 transcripts *Hpse*, *Colq*, and *Agpat4* in *PV*$^+$ neurons in ≥ p56 lumbar DRG of wild type mice. In (*i*) *Hpse* and *Colq* transcripts overlap and are largely restricted to *PV*$^+$ neurons. In (*ii*) expression of *Agpat4* is observed in many neurons in DRG, but within *PV*$^+$ neurons its expression typically overlaps with expression of *Colq*.

e. Expression of cluster 1 transcript *Colq*, in relation to the cluster 5 transcripts *Itga2*, *Chad,* and *Pcdh8*$^+$ in ≥ p56 lumbar DRG of wild type mice.

f, g. Co-expression analysis of various cluster markers within tdT$^+$ pSNs (f), or *PV* expressing neurons (g) per section at lumbar levels (L1-5). In (f) median percentage ± S.E.M. for *Heg1/Pcdh8*: 29.4 ± 4.9%, n=11 sections; for *Colq/Pcdh8*: 23.2 ± 4.3%, n=12 sections; for *Hspe/Pcdh8*: 8.9 ± 2.9%, n=16 sections; for *Agpat4/Pcdh8*: 7.1 ± 2.3%, n=12 sections; for *Nxph1/Pcdh8*: 35.9 ± 5.7%, n=10 sections; for *Nxph1/Cdh13*: 18.0 ± 4.4%, n=15 sections; for *Agpat4/Cdh13*: 37.5 ± 4.8%, n=17 sections. In (g) median percentage ± S.E.M. for *Hspe/PV*: 26.5 ± 2.4%, n=24 sections; for *Fam196a/PV*: 29.7 ± 3.2%, n=24 sections; for *Hspe/Fam196a/PV*: 7.2 ± 1.7%, n=24 sections; for *Agpat4/PV*: 28.9 ± 3.6%, n=24 sections; for *Colq/PV*: 50.0 ± 3.5%, n=24 sections; for *Agpat4/Colq/PV*: 28.9 ± 3.1%, n=24 sections; for *Heg1/PV*: 71.3 ± 3.2%, n=22 sections; for *Grin3a/PV*: 39.0 ± 4.2%, n=22 sections; for *Heg1/Grin3a/PV*: 20.0 ± 2.9%, n=22 sections. Boxes show the median, 25th, and 75th percentile, and whiskers extend to the most extreme data point less than 1.5 times the interquartile range.

Similar data obtained from at least three (a) and two (c-e) biological replicates. Scale 10 μm.

Supplementary Figure 6

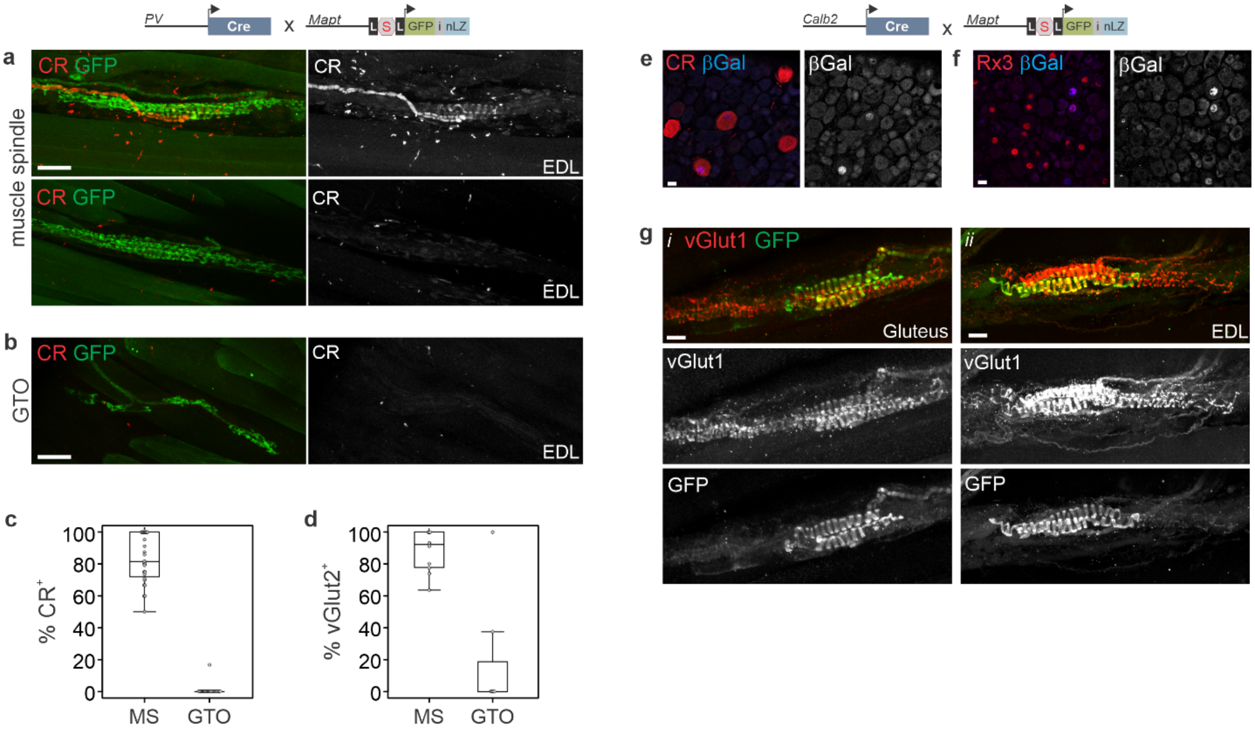

Figure S6. Expression of Calretinin marks group Ia muscle spindle afferents.

a. Colocalization of Calretinin (CR) with GFP in group Ia MS afferents in EDL muscle of p12 *PV:GFP* mice (top panels). In some MS afferents, expression of CR is not observed (bottom panels).

b. CR is not observed in GFP$^+$ GTO afferents in EDL muscle in p12 *PV:GFP* mice.

c. Percentage of MSs and GTOs containing a CR$^+$ sensory terminal across various different muscle targets (see Supplementary Table 1 for details). Median percentage of MSs with a CR$^+$ afferents/muscle 81.5 ± 2.9%, n=28 muscles; median percentage of CR$^+$ GTO afferents/muscle 0.0 ± 0.6%, n=28 muscles. Boxes show the median, 25th, and 75th percentile, and whiskers extend to the most extreme data point less than 1.5 times the interquartile range.

d. Percentage of MSs and GTOs containing a vGlut2$^+$ sensory terminal across various different muscle targets (see Supplementary Table 2 for details). Median percentage of MS with vGlut2$^+$ afferents/muscle 92.1 ± 4.6%, n=10 muscles; median percentage of vGlut2$^+$ GTO afferents/muscle 0.0 ± 14.4%, n=7 muscles. Boxes show the median, 25th, and 75th percentile, and whiskers extend to the most extreme data point less than 1.5 times the interquartile range.

e, f. Genetic labeling of CR positive neurons in DRG using *Calb2:Cre* and *Mapt:lxp-STOP-lxp:GFP-iresNLZ* alleles (hereafter *Calb2:GFP or Calb2:NLZ*). In (e) CR protein colocalizes with the nuclear localized βgalactosidase reporter. Consistent with the notion that CR labels a subset of proprioceptors, the *Calb2:NLZ* reporter is detected in only a small number of Rx3$^+$ neurons (f).

g. Expression of GFP and vGlut1 in MS afferents in hindlimb muscle in p28 *Calb2:GFP* mice. In many muscles, GFP expression is localized to the vGlut1$^+$ MS afferents that occupy the equatorial region of the spindle (*i*), but in some spindles GFP$^+$ axons are not flanked by non-GFP$^+$vGlut1$^+$ afferents (*ii*; a reflection of the heterogeneity with respect to muscle spindle anatomy/innervation patterns).

Similar data obtained from at least three (a, b, e, f) and two (g) biological replicates. Scale 20 μm (e-g) and 50 μm (a, b).

Supplementary Figure 7

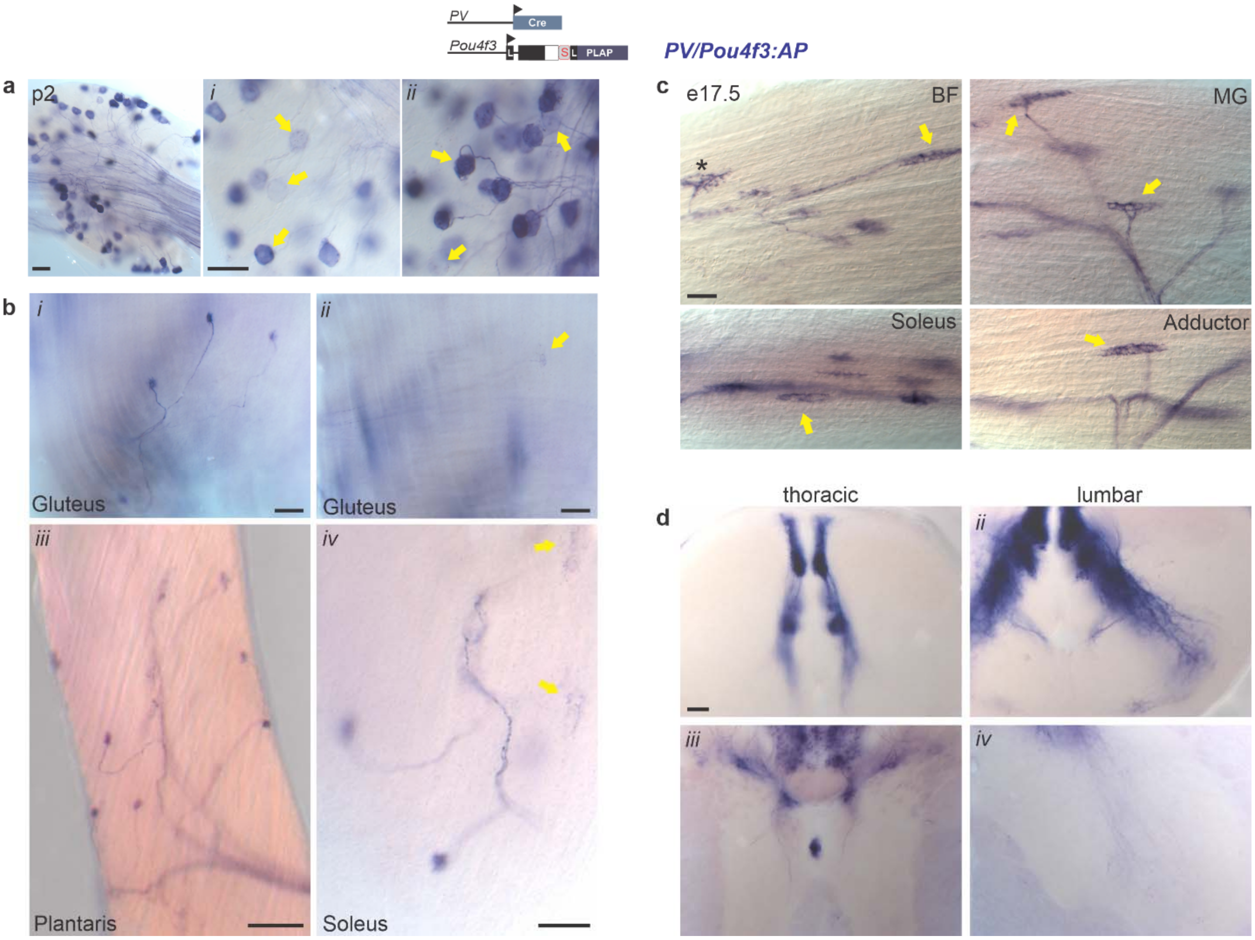

Figure S7. Expression of Pou4f3 in group Ib GTO afferents.

(a-c) Genetic labeling of Pou4f3^{+} sensory neurons in *PV:Cre; Pou4f3:lxp-STOP-lxp:AP* (*PV/Pou4f3:AP*) animals.

(a) PV^{+} and Alkaline Phosphatase^{+} (PV^{+}AP^{+}) sensory neurons in p2 lumbar DRG. Higher magnifications in (*i, ii*) indicate different levels of AP labeling and may suggest a failure to maintain AP expression in some PV neurons.

(b) PV^{+}AP^{+} sensory afferents in p2 Gluteus, Plantaris, and Soleus muscle. In (*i*) AP^{+} GTO afferents and afferent terminals are readily observed, but AP^{+} MS afferents are no longer or just barely visible (arrows in *ii* and *iv*) at this stage.

(c) PV^{+}AP^{+} sensory afferents in e17.5 Biceps Femoris (BF), Gluteus, Medial Gastrocnemius (MG), Soleus, and Adductor muscle. In BF, the distinct sensory ending morphologies of MSs (arrow) and GTOs (asterisk) afferents can be overved.

(d) Spinal axon collaterals of PV^+AP^+ sensory afferents in p2 (*i, ii*) and ≥p56 (*iii, iv*) spinal cord at thoracic (*i, iii*) and lumbar (*ii, iv*) segmental levels. At p2, AP labeling is relatively abundant in all PV^+ neurons and their spinal collaterals (*i, ii*). Consistent with the idea that group Ib GTO afferents primarily terminate on spinal interneurons located within intermediate spinal lamina, by p56, only traces of AP expression can be detected in ventrally projecting neurons (*iii, iv*).

Similar data obtained from at least three (a-c) and two (d) biological replicates. Scale 100 μm (a, b*i, ii, iv*, c, d) and 200 μm (b*iii*).

Supplementary Figure 8

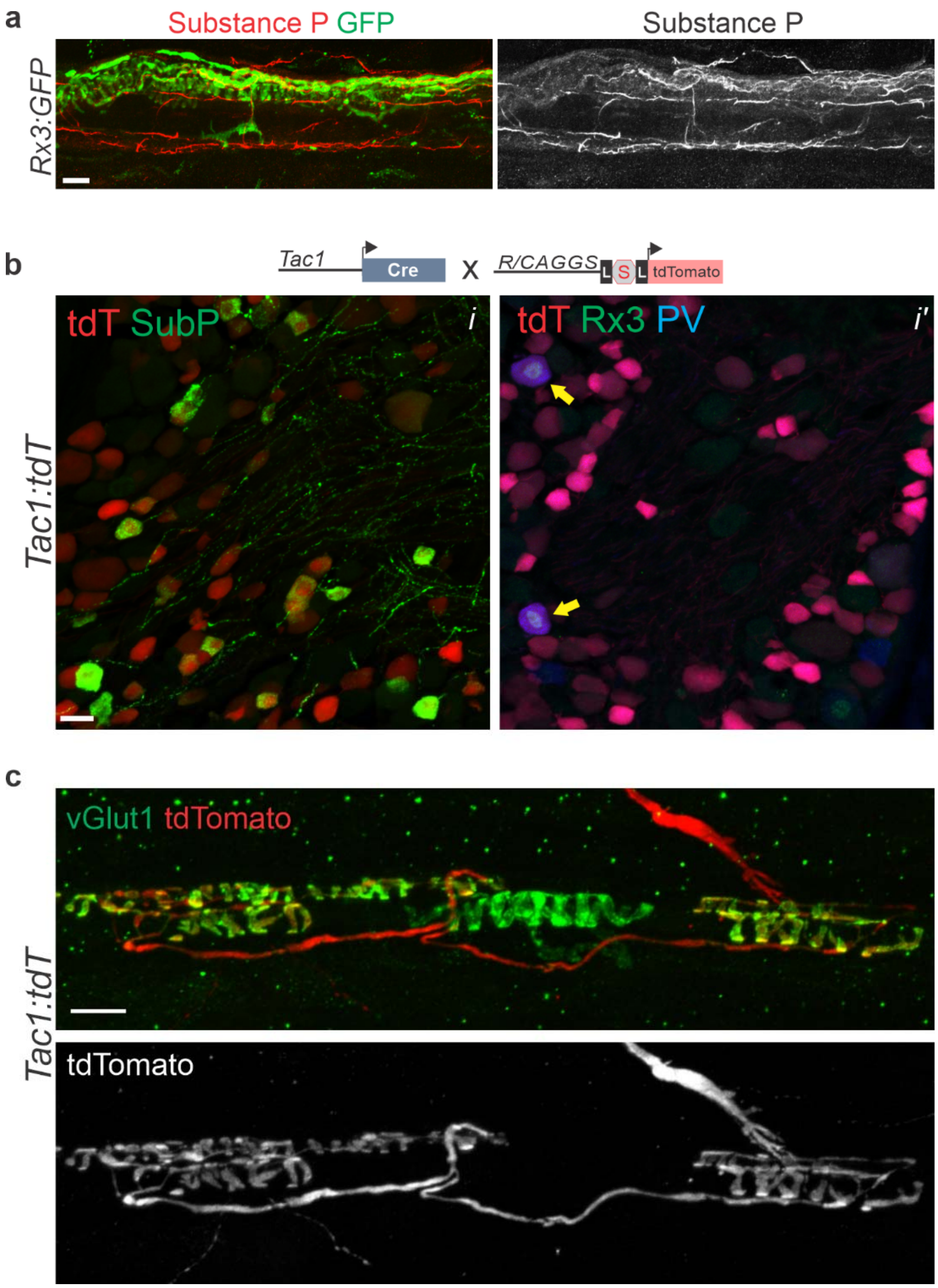

Figure S8. Expression of Substance P in MS afferents.

a. Expression of Substance P and GFP in p12 muscle in *Rx3:GFP* mice. An abundance of Substance P^{+} (Tac1^{+}) sensory terminals in muscle and MS sensory end organs appears to mask low Substance P expression levels in MS.

b. Expression of Substance P and tdT (*i*) and Rx3, PV, and tdT (*ii*) in p21 lumbar DRG of *Tac1:Cre; Ai14:tdTomato* (*Tac1:tdT*) mice. In (*i*) the discrepancy in tdT and Substance P expression provides evidence for a transient activation of *Tac1* in many DRG neurons at earlier developmental stages. Transiently expressing *Tac1* neurons include $PV^{+}Rx3^{+}$ proprioceptors (*ii*), providing a challenge to discern spindle afferents that temporally activated *Tac1* from those that maintain *Tac1* expression.

c. Expression of tdT and vGlut1 in >p28 muscle of *Tac1:Cre; Ai14:tdTomato* (*Tac1:tdT*) mice.

Similar data obtained from one (a) and two (b, c) biological replicates. Scale 20 (a,b) and 50 (c) μm.

Supplementary Figure 9

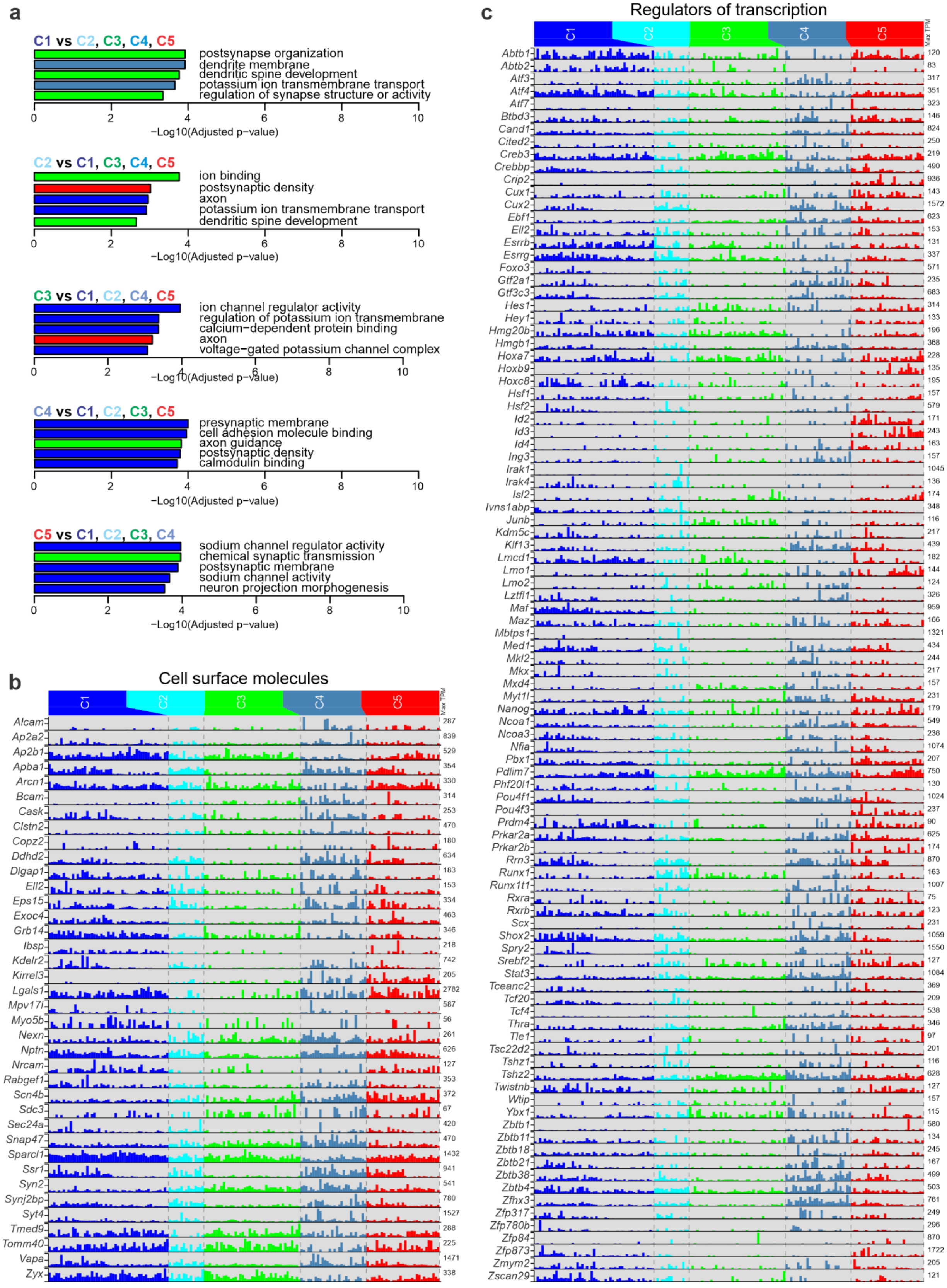

Figure S9. Differential expression of biologically relevant transcripts across molecularly identified pSN subclasses.

a. Go analyses of transcripts differentially expressed between molecularly defined proprioceptor subtypes (one-sided hypergeometric test for statistical enrichment in Gene Ontology Analysis, adjusted for multiple comparisons using Benjamini-Hochberg FDR). Top-5 categories are shown only.

b, c. Barplots showing expression (counts per million, CPM) of cell surface molecules (b) and of transcriptional regulators (c). Genes belonging to each category were obtained from the Panther database, and filtered to include only those with significant differential expression between at least one pair of clusters (FDR p-value < 0.05) and expression in >= 50% of cells within at least one cluster.

Supplementary Figure 10

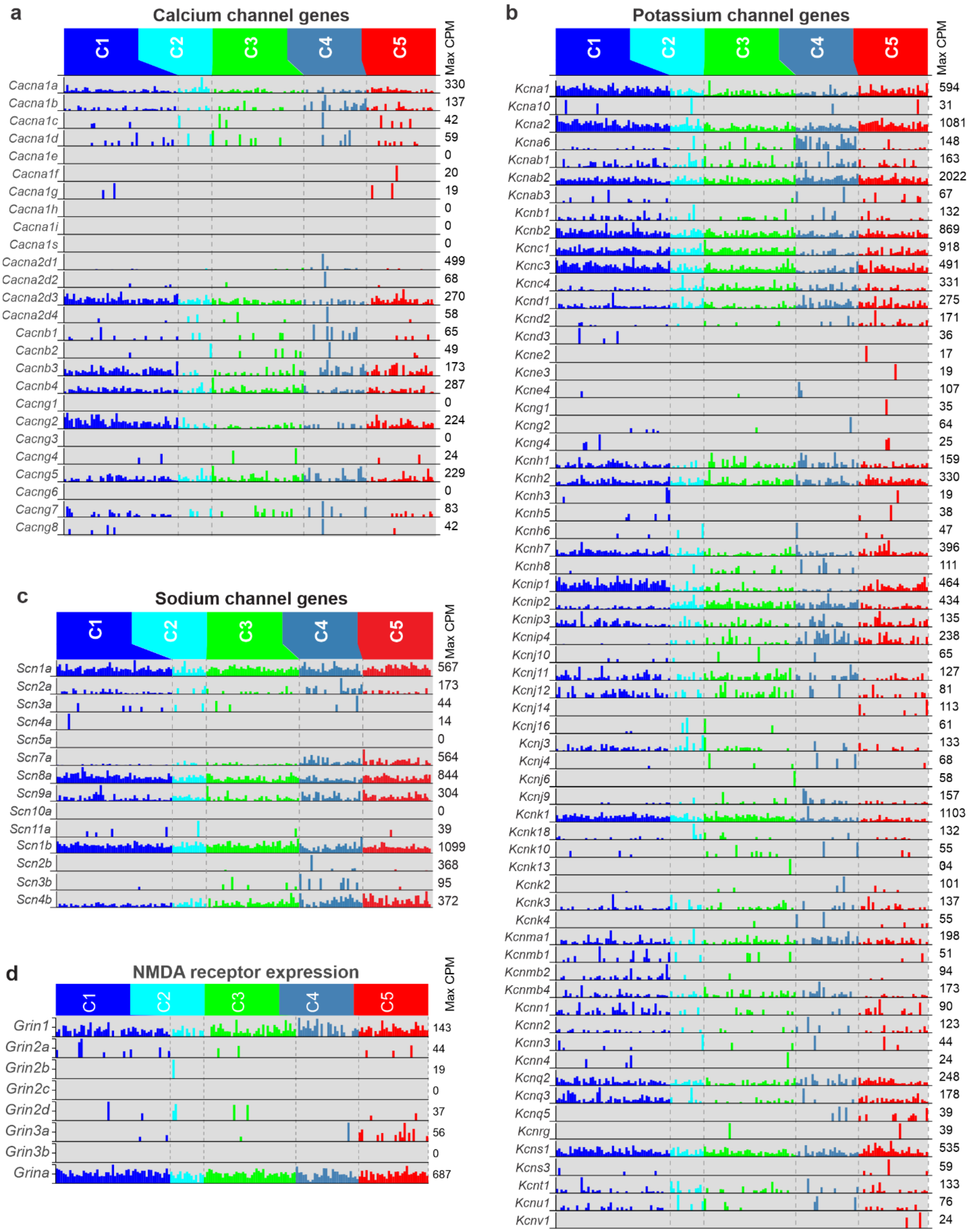

Figure S10. Distribution of ion-channels within muscle pSN subtypes.

a-d. Barplots showing expression (counts per million, CPM) of annotated calcium channel (a), potassium channel (b), sodium channel (c), and NMDA receptor subunit (d) genes across the five putative cell types identified from the clustering of single-cell RNAseq data. Genes belonging to each category were obtained from the Panther database. Potassium channel genes were filtered to include only those genes with expression in at least one cell. Omitted K_v channels (with no appreciable expression in any pSN cluster) are *Kcna3, Kcna4, Kcna5, Kcna7, Kcnc2, Kcne1, Kcne1l, Kcnf1, Kcng3, Kcnh4, Kcnj1, Kcnj5, Kcnj8, Kcnj13, Kcnj15, Kcnk5, Kcnk6, Kcnk7, Kcnk9, Kcnk16, Kcnmb3, Kcnq1, Kcnq4, Kcns2, Kcnt2, and Kcnv2*.

Supplementary Figure 11

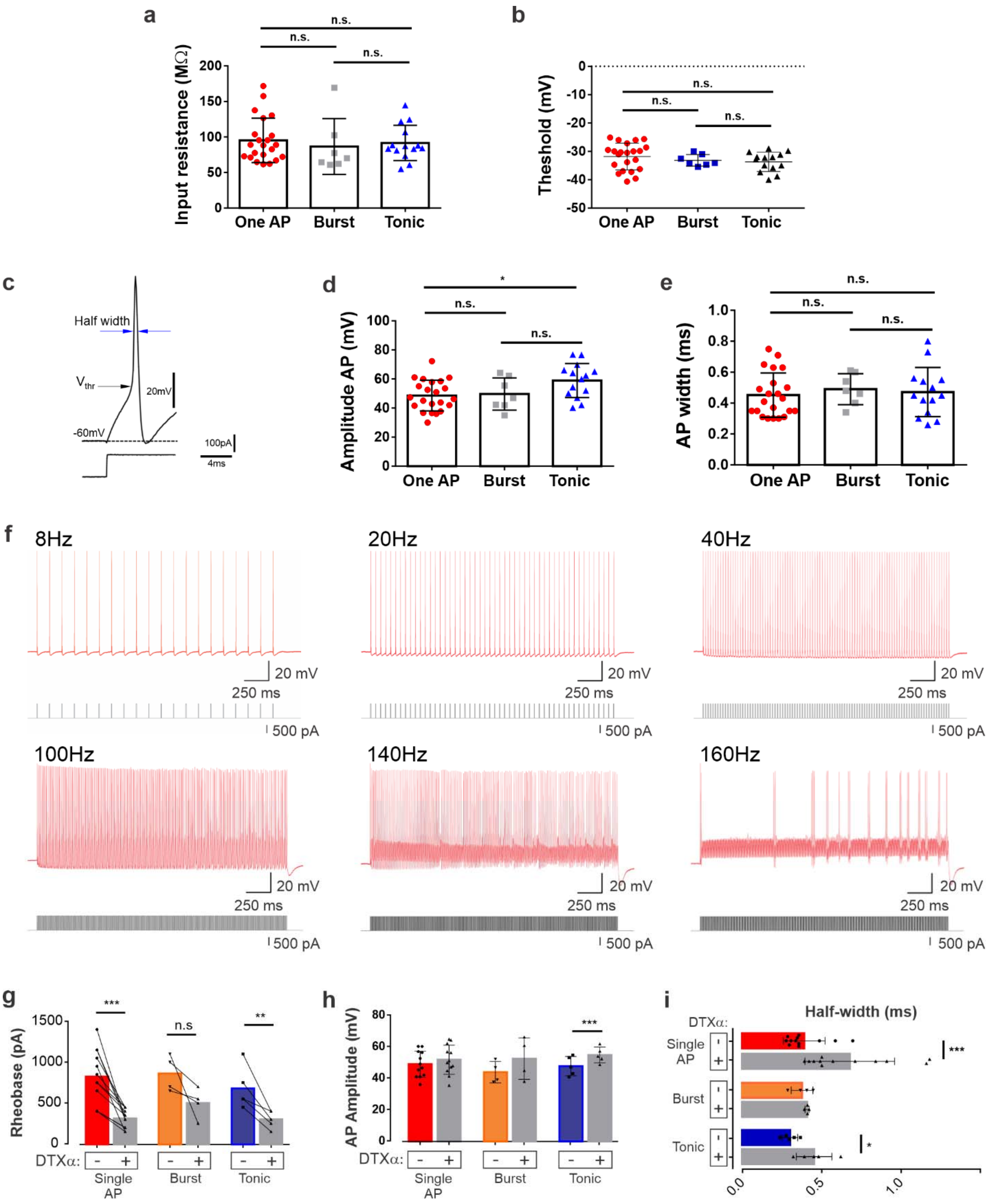

Figure S11. Action potential characteristics, passive membrane properties and ability of proprioceptive neurons to induce action potential firing at different frequencies of stimulation.

a, b. Bar graphs showing Input resistance (a), and Threshold (b), across proprioceptor neurons (One AP, n=22; Burst, n=7; Tonic, n=14). Data expressed as mean ± S.D.; p=0.8053. (One-way ANOVA; Tukey's posthoc test). n.s: not significant.

c. Measurements of the voltage threshold (V_{thr}, black arrow in top trace) and half width of an action potential (blue arrows in top trace) evoked on a proprioceptive neuron following current injection (bottom trace). Dotted line represents holding membrane potential at -60 mV.

d, e. Measurements of amplitude of the action potential (d) and half width of the AP (e) for the three different types of proprioceptors observed in recordings (One AP, n=22; Burst, n=7; Tonic, n=14). Data expressed as mean ± S.D.; Amplitude AP p=0.0262; AP width p=0.8079 (One-way ANOVA; Tukey's *posthoc* test). n.s.: not significant.

(f) Representative voltage responses (red traces on top) following different frequencies of a small step of current injection (black traces on bottom) in the same neuron. Frequencies range from 8 Hz to 160 Hz.

(g-i) Bar graphs showing changes in Rheobase (g), amplitude of AP (h) and half-width of AP (i), before (in color) and during 20nM DTXα (grey) for the three different physiological types of proprioceptors (One AP, n=12; Burst, n=5; Tonic, n=4). Each dot represents a single neuron. P values for rheobase (Single AP, p<0.001. Burst p=0.0596. Tonic, p=0.0075); AP amplitude (Single AP, p=0.112. Burst p=0.1720. Tonic, p=0.0006); Half-width (Single AP, p<0.001. Burst p=0.2616. Tonic, p=0.0150) (Paired-t-test, one tail). n.s.: not significant.

Supplementary Figure 12

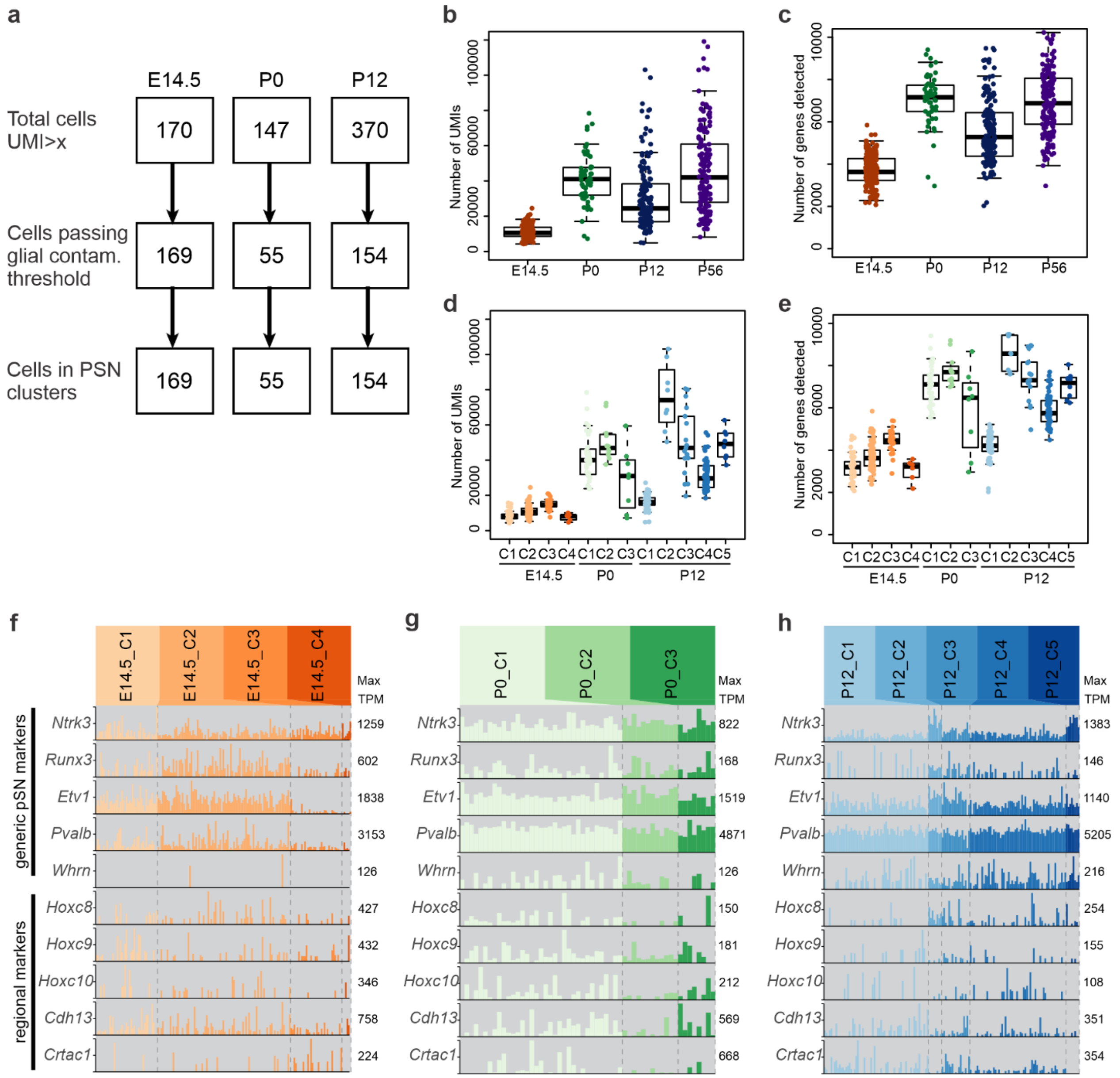

Figure S12. Analysis of developmental scRNAseq data sets.

a. Overview of the number of starting and filtered cells profiled using the Plate-Seq method (see Methods) for three developmental time points. From the initial plate wells, cells were assessed for minimal cross-talk with non-neuronal transcripts, chiefly satellite cell genes such as Mbp (see Methods). Of the cells passing the contamination threshold, only those that ultimately belonged to clusters with >=5 cells were retained in the final step.

b, c. Boxplots showing the number of (b) Unique Molecular Identifiers (UMIs) and (c) genes detected per cell at each time point (number of cells: e14.5, n=169; p0, n=55; p12, n=154; p56, n=166). Points for

each individual cell are overlaid on the boxplots. Boxes indicate medians and 25th/75th percentiles, and whiskers extend to the furthest point less than 1.5 standard deviations from the mean.

d, e. Boxplots showing the number of (d) Unique Molecular Identifiers (UMIs) and (e) genes detected per cell, subdivided by cluster (number of cells at e14.5, C1: 41, C2: 88, C3: 34, C4: 6; p0, C1: 35, C2: 12, C3: 8; p12, C1: 63, C2: 8, C3: 17, C4: 58, C5: 8). Points for each individual cell are overlaid on the boxplots. Boxes indicate medians and 25th/75th percentiles, and whiskers extend to the furthest point less than 1.5 standard deviations from the mean.

f, h. Barplots showing expression (counts per million, CPM) of previously reported pSN and regional markers in the adult mouse, with individual cells arranged by cluster at each time point. The maximum CPM for each gene is reported on the right hand side.

Supplementary Figure 13

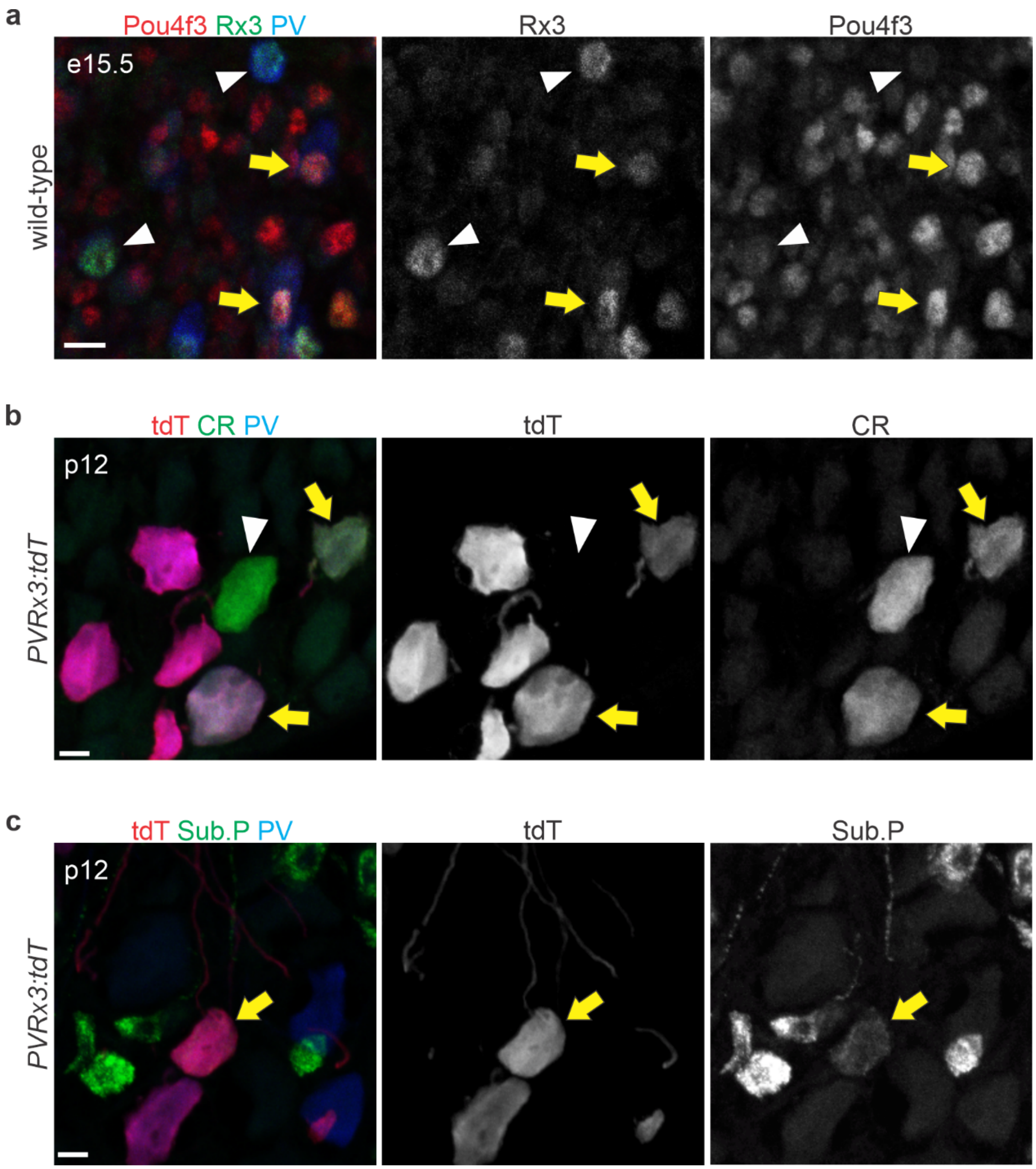

Figure S13. Expression pattern of newly identified MS and GTO afferent molecular markers in DRG.

a. Expression of Pou4f3, Rx3, and PV in e15.5 lumbar DRG in wild type mice. Arrows indicate $Pou4f3^{+}PV^{+}Rx3^{+}$ pSNs; arrowheads indicate $PV^{+}Rx3^{+}$ pSNs with little or no Pou4f3 expression.

b. Expression of CR, PV and tdT in p12 lumbar DRG in *PVRx3:tdT* mice. Arrows indicate CR, $PV^{+}tdT^{+}$ pSNs; arrowheads indicate $Calretinin^{+}tdT^{off}$ non-pSNs.

c. Expression of Sub. P, PV and tdT in p12 lumbar DRG in *PVRx3:tdT* mice. Arrow indicates $SubP^+PV^+tdT^+$ pSN.

Similar data obtained from at least three biological replicates. Scale 10 μm.

Supplementary Figure 14

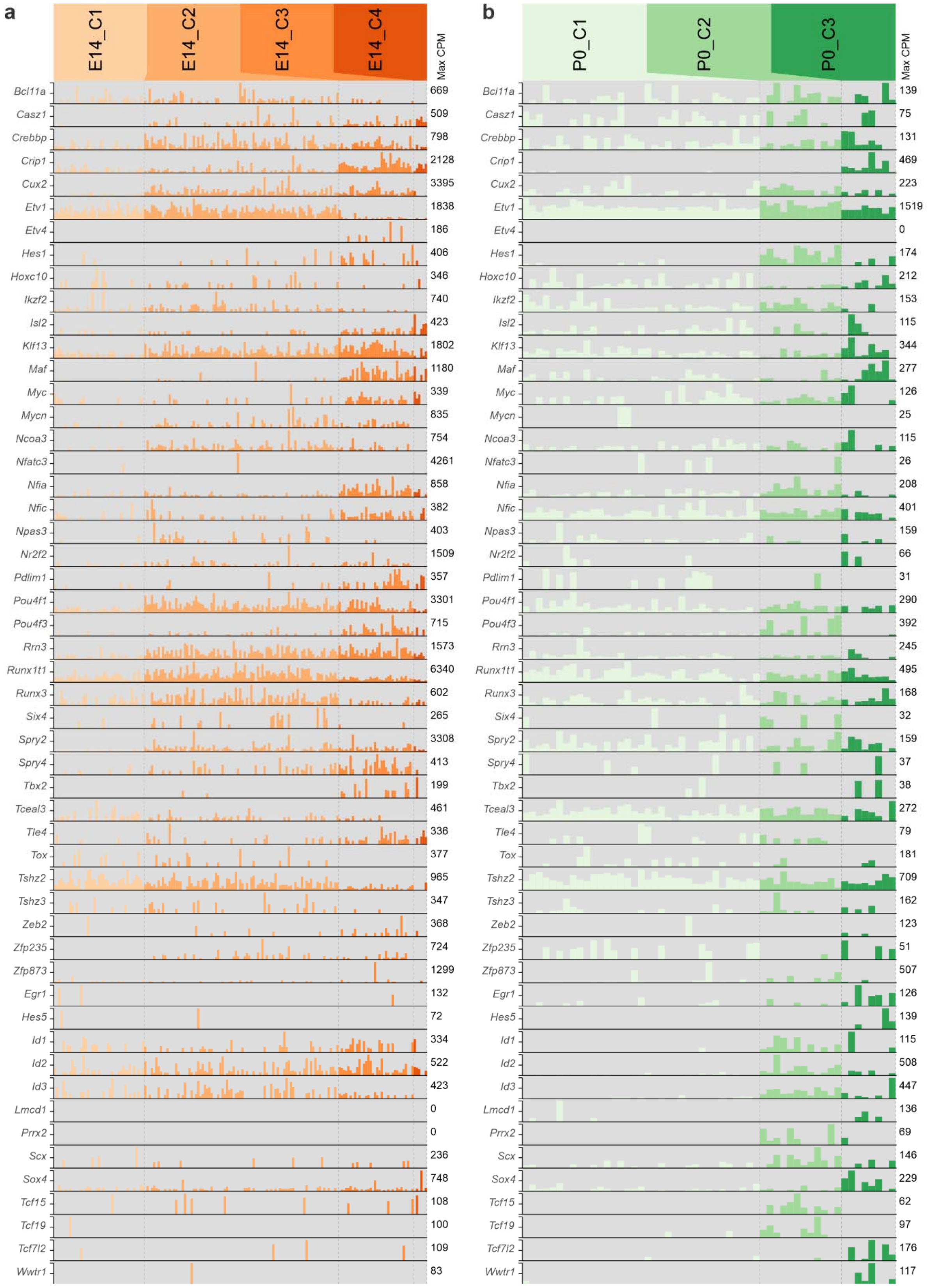

Figure S14. Transcription factor expression across e14.5 and p0 pSN molecular subtypes.

a, b. Barplots showing expression (transcripts per million, CPM) of transcriptional regulators in cells from e14 (a) and P0 (b). Genes were obtained from the Panther database, and filtered to include only those with significant differential expression between at least one pair of clusters within a time point (FDR p-value < 0.05; two-sided test using EdgeR) and expression in at least 3 cells in one cluster.

Supplementary Figure 15

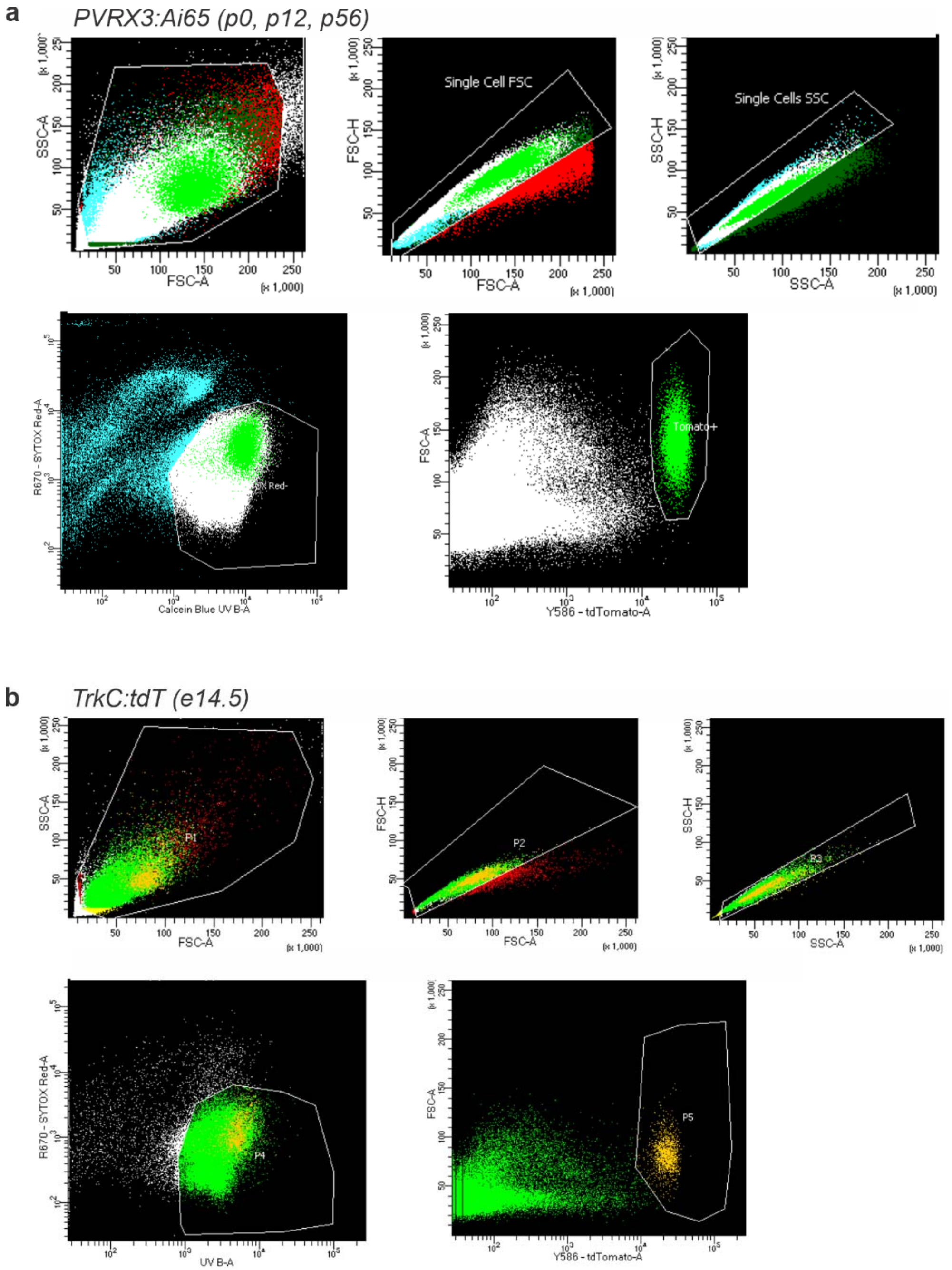

Figure S15. FACS gating strategy using in experiments.

a, b. Gating strategy for *PVRx3:Ai65*⁺ (p0, p12, p56) (a) and TrkC:tdT+ (e14.5) (b) proprioceptors (in sequence). Cells were selected from cellular debris using Forward Scatter (FSC) and Side Scatter (SSC), as customary. Single cells were selected from doublets using FSC-Area and FSC-Height, and SSC-Area and SSC-Height. Healthy cells (~90% of the single cell population) were defined by bright Calcein Blue stain

and negativity for Sytox Red. tdTomato cells (~2-3% of healthy cells for *PVRx3:Ai65*; 2.5-3.5% of healthy cells for *TrkC:tdT*) were defined by plotting tdTomato against the FSC, which typically yielded a clearly discernible cell population at high tdT fluorescent intensity.

Supplementary Table 1. Prevalence of Calretinin+ afferents associated with MS and GTO sensory endings across various muscle targets.

Muscle	I.D.[1]	#MS[2]	CR+	%CR+	tdT+MS[3]	#GTO[2]	CR+	% CR+
Adductor	2	15	9	60.0	0	4	0	0.0
	3	10	8	80.0		1	0	0.0
Biceps Femoris		nd	nd	nd	3			
Gluteus	1	15	11	73.3	2	4	0	0.0
	2	16	13	81.3		18	0	0.0
	3	15	12	80.0		9	0	0.0
	5	18	18	100.0		1	0	0.0
EDL	1	10	10	100.0	12	2	0	0.0
	2	10	10	100.0	12	2	0	0.0
	3	11	10	90.9		0	0	0.0
	4	8	7	87.5		2	0	0.0
Soleus	1	10	7	70.0	0	5	0	0.0
	2	4	2	50.0	0	3	0	0.0
	4	5	3	60.0		3	0	0.0
Plantaris	2	4	3	75.0	0	6	0	0.0
	3	4	3	75.0	0	6	0	0.0
	4	5	5	100.0		6	1	16.7
ECR	2	15	15	100.0	11	3	0	0.0
	3	14	14	100.0	10	1	0	0.0
	4	7	6	85.7		4	0	0.0
dFL flexors	1	17	12	70.6	3	3	0	0.0
	2	21	20	95.2		8	0	0.0
	3	16	16	100.0		1	0	0.0
dFL extensors	2	19	20	105.3	5	7	0	0.0
	3	8	8	100.0	9	1	0	0.0
Bodywall	1	3	2	66.7	0	0	0	0.0
	2	19	15	78.9	0	1	0	0.0
	3	11	9	81.8		1	0	0.0
Axial	2	12	8	66.7	0	0	0	0.0
**TOTAL**	28	322	276	85.7		102	1	1.0

Notes: ***1)*** Number indicates animal identity in which MS and GTO innervation was examined; identical numbers indicate analyzed muscle was derived from the same animal (excluding all tdT+ data). ***2)*** Number of MSs and GTOs based on tdT expression in *PVRx3:tdT* animals; MSs or GTOs too deep in the tissue to clearly delineate were not included in counts. ***3)*** total number of tdT+ spindles observed in muscles obtained from *Calb2:Cre;Tau:mGFP or Calb2:Cre;Rx3:FlpO;Ai65:tdT* animals. For typical numbers of MS in these muscles see column 2. tdT+ GTO sensory endings were not observed in any of the muscles examined. Abbreviations: Extensor Digitorum Longus (EDL), Extensor Carpi Radialis (ECR), distal forelimb (dFL).

Supplementary Table 2. Prevalence of vGlut2$^+$ afferents associated with MS and GTO sensory endings across various muscle targets.

Muscle	**I.D.**[1]	**#MS**[2]	**"Ia" vGlut2$^+$**	**%vGlut2$^+$**	**#GTO**[2]	**vGlut2$^+$**	**%vGlut2$^+$**
EDL	1	7	7	100.0	3	0	0.0
Gluteus	1	15	14	93.3	10	0	0.0
Soleus	1	11	7	63.6	1	0	0.0
Plantaris	1	2	2	100.0	5	0	0.0
Adductor	1	9	7	77.8	nd	nd	nd
Axial	1	3	3	100.0	2	0	0.0
Bodywall[4]	1	10	8	80.0	nd	nd	nd
ECR	1	11	10	90.9	1	1[3]	100.0
dFL extensors	1	10	11	110.0	nd	nd	nd
dFL flexors	1	19	14	73.7	8	3[3]	37.5
**TOTAL**		97	83	85.6	30	4	13.3

Notes: ***1)*** Number indicates animal identity in which MS and GTO innervation was examined; identical numbers indicate analyzed muscle was derived from the same animal. ***2)*** Number of MSs and GTOs based on tdT expression in *PVRx3:tdT* animals; spindles too deep in the tissue to clearly delineate were not included in counts. ***3)*** Unclear if expression localized to myelinating Schwann cells or afferent fiber. ***4)*** Also contained 4 vGlut2$^+$ endings with a group II appearance. Abbreviations: Extensor Digitorum Longus (EDL), Extensor Carpi Radialis (ECR), distal forelimb (dFL), not determined (nd).

Supplementary Table 3. Prevalence of tdT$^+$ afferents associated with MS and GTO sensory endings across muscle targets in *Tac1:Cre;Ai14:tdT* animals.

muscle	#MS[1]	#MS afferents[2]	#Ia	#II	#tdT$^+$ Ia	% tdT$^+$ Ia	#tdT$^+$ II	% tdT$^+$ II	ratio tdT$^+$ II/Ia	#GTO[1]	#tdT$^+$	% tdT$^+$ GTO
Gluteus	22	52	22	30	14	63.6	27	90	1.4	7	0	0
EDL	7	17	7	10	6	85.7	10	100	1.2	5	5	100
Bodywall	6	10	6	4	6	100.0	4	100	1.0	1	0	0
Intercostal	14	30	14	16	1	7.1	6	37.5	5.3	0	0	0

Notes: ***1)*** Number of MSs and GTOs based on vGlut1 expression; spindles too deep in the tissue to clearly delineate were not included in counts. ***2)*** Exact number of afferents difficult to assess due to terminal branching. Abbreviations: Extensor Digitorum Longus (EDL).
