# Supplementary material for: Molecular correlates of muscle spindle and Golgi tendon organ afferents
Source: Nat Commun. 2021 Mar 1;12:1451. doi: 10.1038/s41467-021-21880-3 (PMC7977083; doi:10.1038/s41467-021-21880-3)
Supplement: Supplementary file 3 — Reporting Summary [file 41467_2021_21880_MOESM3_ESM.pdf]

nature research

Corresponding author(s): Joriene C. de Nooij

Last updated by author(s): Jan 19, 2021

Reporting Summary

Nature Research wishes to improve the reproducibility of the work that we publish. This form provides structure for consistency and transparency in reporting. For further information on Nature Research policies, see Authors & Referees and the Editorial Policy Checklist .

Statistics

For all statistical analyses, confirm that the following items are present in the figure legend, table legend, main text, or Methods section.

n/a	Confirmed	
☐	☒	The exact sample size (*n*) for each experimental group/condition, given as a discrete number and unit of measurement
☐	☒	A statement on whether measurements were taken from distinct samples or whether the same sample was measured repeatedly
☐	☒	The statistical test(s) used AND whether they are one- or two-sided<br>*Only common tests should be described solely by name; describe more complex techniques in the Methods section.*
☒	☐	A description of all covariates tested
☒	☐	A description of any assumptions or corrections, such as tests of normality and adjustment for multiple comparisons
☐	☒	A full description of the statistical parameters including central tendency (e.g. means) or other basic estimates (e.g. regression coefficient) AND variation (e.g. standard deviation) or associated estimates of uncertainty (e.g. confidence intervals)
☐	☒	For null hypothesis testing, the test statistic (e.g. *F*, *t*, *r*) with confidence intervals, effect sizes, degrees of freedom and *P* value noted<br>*Give P values as exact values whenever suitable.*
☒	☐	For Bayesian analysis, information on the choice of priors and Markov chain Monte Carlo settings
☒	☐	For hierarchical and complex designs, identification of the appropriate level for tests and full reporting of outcomes
☐	☒	Estimates of effect sizes (e.g. Cohen's *d*, Pearson's *r*), indicating how they were calculated

Our web collection on statistics for biologists contains articles on many of the points above.

Software and code

Policy information about availability of computer code

Data collection	Software/Code used for data acquisition: Zen 3.0 (black-edition), Zeiss (https://www.zeiss.com); Leica Application Suite X, Leica Microsystems (https://www.leica-microsystems.com); Clampex 10.1 (Multiclamp 700B commander), Molecular Devices (https://www.moleculardevices.com); FACSDiva v8.0.2, BD Biosciences (https://www.bdbiosciences.com).
Data analysis	Software/Code used for data analysis: Photoshop CC2017 (count tool), Adobe (https://www.adobe.com); Sigmaplot v14, Systat systems (https://systatsoftware.com); R v3.5.0. (Edge R, hicat R, and limma R), R-project (https://www.r-project.org); GraphPad Prism 6, GraphPad Inc. (https://www.graphpad.com); Clampfit 11.1, Molecular Devices (https://www.moleculardevices.com); Origin8, Origin lab (www.originlab.com)

For manuscripts utilizing custom algorithms or software that are central to the research but not yet described in published literature, software must be made available to editors/reviewers. We strongly encourage code deposition in a community repository (e.g. GitHub). See the Nature Research guidelines for submitting code & software for further information.

Data

Policy information about availability of data

All manuscripts must include a data availability statement. This statement should provide the following information, where applicable:

- Accession codes, unique identifiers, or web links for publicly available datasets
- A list of figures that have associated raw data
- A description of any restrictions on data availability

All sequencing data is available through the NCBI GEO database (accession #GSE162263); A web based searchable database is also available at https://vmenon.shinyapps.io/proprioceptors_scrnaseq.

Field-specific reporting

Please select the one below that is the best fit for your research. If you are not sure, read the appropriate sections before making your selection.

- [x] Life sciences
- [ ] Behavioural & social sciences
- [ ] Ecological, evolutionary & environmental sciences

For a reference copy of the document with all sections, see nature.com/documents/nr-reporting-summary-flat.pdf

Life sciences study design

All studies must disclose on these points even when the disclosure is negative.

Sample size

Number of neurons for single cell RNAseq analysis were set on the basis of obtaining at least 200 proprioceptors from adult mouse, which results in a >99% probability of obtaining at least 10 cells of a cell type present at 10% or more in the proprioceptor population.
For validation of single cell RNAseq data, sample sizes were chosen to allow validation of reproducibility of the results and were based on the assumption that three independent samples customary suffice to test reproducibility in wild type conditions. Total counts of neurons were based on the number of image sections processed for each experiment (at least 6/exp).

Data exclusions

For downstream analysis, only cells that did not pass QC (sufficient transcripts detected, minimal contamination with satellite cell genes) were excluded, as noted in the manuscript. These QC metrics for sufficient transcript detection (number of transcripts detected) and contamination (percent of reads coming from non-neuronal cell types) were based on prior single-cell RNA-seq studies in the field that have established benchmarks for usable single-cell data.

Replication

As with most single-cell RNA-seq studies, no replication of the single-cell data was performed. The single-cell RNA-seq studies are meant to generate hypothesis, as opposed to validate one. As a result, the replication/validation aspect of this study was done through an orthogonal method (multiplexed FISH and immunohistochemistry), which was replicated.

Randomization

Randomization was not relevant because there was no comparison between experimental and control groups (all data is from wild type animals), and none of the data generated is ordinal in any way. The single-cell RNA-seq protocol, because it relies on sorting from a cell suspension, already randomizes the selection of cells from the population.

Blinding

There was no comparison between experimental and control groups in these studies; Blinding was not relevant to these studies.

Reporting for specific materials, systems and methods

We require information from authors about some types of materials, experimental systems and methods used in many studies. Here, indicate whether each material, system or method listed is relevant to your study. If you are not sure if a list item applies to your research, read the appropriate section before selecting a response.

Materials & experimental systems

n/a	Involved in the study
[ ]	[x] Antibodies
[x]	[ ] Eukaryotic cell lines
[x]	[ ] Palaeontology
[ ]	[x] Animals and other organisms
[x]	[ ] Human research participants
[x]	[ ] Clinical data

Methods

n/a	Involved in the study
[x]	[ ] ChIP-seq
[ ]	[x] Flow cytometry
[x]	[ ] MRI-based neuroimaging

Antibodies

Antibodies used

Rabbit anti-Calbindin D-28k (1:2,000) Swant cat# CB-38a; RRID: AB_10000347
Rabbit anti Calretinin (Calb2; 1:2,000) Swant cat# 7699/4; RRID:AB_2313763
Guinnee pig anti-tdTomato Wu et al., 2019 N/A
Rabbit anti-dsRED (1:1,000) Clontech cat# 632496; RRID:AB_10013483
Rabbit anti-GFP (1:1000) ThermoFisher A-11122; RRID:AB_221569
Sheep anti-GFP (1:500) AbD Serotec cat# 4745-1051
Rat anti-Troma1 (1:100) DSHB cat# TROMA-1; RRID:AB_531826
Rat anti-Substance P (1:200) Santa Cruz Biotechnology cat# sc-21715; RRID:AB_628299
Rabbit anti-Runx3 (1:50,000) Kramer et al., 2006 N/A
Guinnee pig anti-Runx3 (1:16,000) Chen et al., 2006 N/A
Chicken anti-Parvalbumin (1:30,000) de Nooij et al., 2013 N/A
Guinnee pig anti-Islet 1 (1:20,000) Dasen et al., 2005 N/A

Rabbit anti-vGlut1 (1:16,000) Demireva et al., 2011 N/A
Rabbit anti-VGlut2 (1:500) Synaptic Systems cat#135403; RRID:AB_887883
Rabbit S-100 (1:400) Agilent cat# Z0311; RRID:AB_10013383
Chicken anti-Brn3c (1:50) Goel et al., 2019 N/A
chicken anti-b-galactosidase (1:5,000) Abcam cat#AB9361; RRID:AB_307210
rabbit anti-PCDH8 (1:250) Millipore cat# ABT153

Fluorophore-conjugated secondary antibodies generated in donkey (Jackson Immuno Research Laboratories) were used at 1:1000 (FITC) or 1:500 (Cy3, Cy5)

Validation

Validation of antibodies was based on 2) examination of expression (absence in mutant animals, or positive correlation with known cellular markers), 2) comparison with RNA-in situ data, or 3) prior evidence in the literature. Specific information for each antibody is described in the product information as listed on the suppliers website or in the references listed with individual antibodies.

Animals and other organisms

Policy information about studies involving animals; ARRIVE guidelines recommended for reporting animal research

Laboratory animals

Mouse: Runx3:FlpO this paper; experimental animals using this allele included both sexes and were used from p0-p56
Mouse: PV:Cre Hippenmeyer et al., 2005; Jackson labs # 017320; experimental animals using this allele included both sexes and were used from p0-p56
Mouse: Ai65D Madison et al., 2015; Jackson labs # 021875; experimental animals using this allele included both sexes and were used from p0-p56
Mouse: RCE:FRT Sousa et al., 2009 Jackson labs # 32038-JAX; adult animals of both sex were used for experiments
Mouse: Ai14 Madison et al., 2010 Jackson labs # 007914; adult animals of both sex were used for experiments
Mouse: Mapt:eGFP-nLZ Hippenmeyer et al., 2005 N/A; adult animals of both sex were used for experiments
Mouse: TrkC:tdTomato Bai et al., 2015; Jackson labs # 030292; embryonic animals (e14.5) of unspecified sex were used in experiments
Mouse: Calb2-IRES-Cre Taniguchi et al., 2011; Jackson labs # 010774; adult animals of both sex were used for experiments
Mouse: Tac1-IRES2-Cre-D Harris et al., 2014 Jackson labs # 021877; adult animals of both sex were used for experiments
Mouse: Pou4f3tm1.1Nat Badea et al., 2011 Jackson labs # 010560; experimental animals using this allele included both sexes and were used from p0-p56

All animals were housed under ambient temperature conditions, ranging between 69-74oF, with 40-60% humidity, and were kept on a 12:12 light:dark cycle. Animals were given ad lib access to water and food.

Wild animals

Study did not involve wild animals

Field-collected samples

Study did not involve field collected samples

Ethics oversight

All experiments were performed according to National Institutes of Health guidelines and approved by the Institutional Animal Care and Use Committee of Columbia University

Note that full information on the approval of the study protocol must also be provided in the manuscript.

Flow Cytometry

Plots

Confirm that:

- [ ] The axis labels state the marker and fluorochrome used (e.g. CD4-FITC).
- [ ] The axis scales are clearly visible. Include numbers along axes only for bottom left plot of group (a 'group' is an analysis of identical markers).
- [ ] All plots are contour plots with outliers or pseudocolor plots.
- [x] A numerical value for number of cells or percentage (with statistics) is provided.

Methodology

Sample preparation

DRG from adult (p>56), adolescent (p12), neonatal (p0), and embryos (e14.5) of either sex were dissected (1 hour maximum) in ice-cold Hank's balanced salt solution (HBSS) and collected, on ice, in HBSS supplemented with 0.75% horse serum (HS). The total number of animals (of either sex) used for experiments was six for adult samples, four for p12, six for p0, and two for e14.5. Following dissection, DRGs were centrifuged at low speed and washed once with ice cold HBSS prior to dissociation though enzymatic digestion using Papain followed by Collagenase/Dispase35,59. Papain digestion step consisted of 3 ml HBSS with ~16 units/ml Papain (Worthington), 0.83 mM L-Cysteine, 0.42 mM EDTA, and 20 units DNAse I/ml (Roche). Collagenase/Dispase digestion was with 3 ml HBSS containing 1,066 units/ml Collagenase IV (Worthington), 4 units/ml Dispase (Worthington), and 20 units/ml DNase I (Roche). Duration of digestion incubation times was 16 min for adult, 12 min for p12, 10 minutes for p0 and e14.5 (for both enzyme digestion steps). Solutions were exchanged by a low speed centrifuge step (4' at 800 rpm in table top Eppendorf centrifuge) and aspiration. After Collagenase/Dispase digestion, enzyme solution was replaced with 500l HBSS

supplemented with 20% HS and 20 units DNAse I, and DRG/cell suspension was dissociated by slow mechanical trituration using a 200 ul pipetman (~50 times). Following dissociation, cell suspension was incubated with 2M Calcein Blue (CellTrace™ Calcein Blue, AM; Invitrogen) for 15 minutes at room temperature. After Calcein Blue labeling, cells were spun down (4' at 800 rpm) and resuspended in 500l sorting solution (HBBS, 1% HS, 20 units/ml DNAse I) supplemented with 0.01M Sytox red (SYTOX™ Red Dead Cell Stain; Invitrogen). Just prior to FACS, dissociated cells were passed through 40-70m gauze filters to clear remaining cellular aggregates.

Instrument

Becton Dickinson FACSAria (SORP model, 5-laser, 20 parameter) using 586/15 (tdTomato), 450/50 (Calcein Blue), and 670/30 (Sytox red) filter sets. A 130 um nozzle was used for p0, p10 and adult proprioceptors. For e14.5 embryonic neurons a 100uM nozzle was used.

Software

FACSDiva v8.0.2

Cell population abundance

Cells were sorted as single cells in 96-well plates. Viable tdTomato positive sensory neurons were isolated using 586/15 (tdTomato), 450/50 (Calcein Blue), and 670/30 (Sytox red) filter sets. Percentage of viable cells in PV:Cre;Rx3FlpO; Ai65 sorts (p0, p12, p56) was typically ~90%; and percentage of viable tdT neurons ranged between 2-3%. Percentage of viable cells in TrkC:tdTomato sorts (e14.5) was typically ~90%; and percentage of viable tdT neurons ranged between 2.5-3%.

Gating strategy

Cells were selected from cellular debris, as customary, using Forward Scatter (FSC) and Side Scatter (SSC). Single cells were selected from doublets using FSC-Area and FSC-Height, and SSC-Area and SSC-Height. Healthy cells (~90% of the single cell population) were defined by bright Calcein Blue stain and negativity for Sytox Red. tdTomato cells were defined by plotting tdTomato against the FSC, which typically yielded a clearly discernible cell population at higher tdT fluorescent intensity.

☒ Tick this box to confirm that a figure exemplifying the gating strategy is provided in the Supplementary Information.
